# MED31/437: A Web-based Diabetes Management System: DiabNet

**DOI:** 10.2196/jmir.1.suppl1.e68

**Published:** 1999-09-19

**Authors:** N Zhao, A Roudsari, E Carson

**Affiliations:** ^1^City UniversityLondonUK

**Keywords:** Web-based, ActiveX, Diabetes Management, Decision-Making, Mobile Computing

## Abstract

**Introduction:**

A web-based system (DiabNet) was developed to provide instant access to the Electronic Diabetes Records (EDR) for end-users, and real-time information for healthcare professionals to facilitate their decision-making. It integrates portable glucometer, handheld computer, mobile phone and Internet access as a combined telecommunication and mobile computing solution for diabetes management. Methods:

Active Server Pages (ASP) embedded with advanced ActiveX controls and VBScript were developed to allow remote data upload, retrieval and interpretation. Some advisory and Internet-based learning features, together with a video teleconferencing component make DiabNet web site an informative platform for Web-consultation.

**Results:**

The evaluation of the system is being implemented among several UK Internet diabetes discussion groups and the Diabetes Day Centre at the Guy's & St. Thomas' Hospital. Many positive feedback are received from the web site demonstrating DiabNet is an advanced web-based diabetes management system which can help patients to keep closer control of self-monitoring blood glucose remotely, and is an integrated diabetes information resource that offers telemedicine knowledge in diabetes management.

**Discussion:**

In summary, DiabNet introduces an innovative online diabetes management concept, such as online appointment and consultation, to enable users to access diabetes management information without time and location limitation and security concerns.

